# Crystal structure of 2-phenyl­ethanaminium 3-carb­oxy­prop-2-enoate

**DOI:** 10.1107/S2056989015014292

**Published:** 2015-08-06

**Authors:** N. Swarna Sowmya, S. Sampathkrishnan, R. Akilan, G. Chakkaravarthi, R. Mohan Kumar

**Affiliations:** a Department of Applied Physics, Sri Venkateswara College of Engineering, Chennai 602 117, India; b Department of Physics, Aksheyaa College of Engineering, Kancheepuram 603 314, India; c Department of Physics, CPCL Polytechnic College, Chennai 600 068, India; d Department of Physics, Presidency College, Chennai 600 005, India

**Keywords:** crystal structure, mol­ecular salt, aminium, 3-carb­oxy­prop-2-enoate, hydrogen bonding

## Abstract

The title mol­ecular salt, C_8_H_12_N^+^·C_4_H_3_O_4_^−^, crystallized with two independent cations and anions in the asymmetric unit. The ethanaminium side chains of the cations exhibit *anti* conformations [C—C—C—N torsion angles = 176.5 (3) and −179.4 (3)°]. In the crystal, N—H⋯O and C—H⋯O hydrogen bonds connect adjacent anions and cations, and , O—H⋯O hydrogen bonds connect adjacent anions, generating sheets parallel to (001).

## Related literature   

For the crystal structures of related compounds, see: Ambalatharasu *et al.* (2014 ▸); Lejon *et al.* (2006 ▸); Smith *et al.* (2003 ▸).
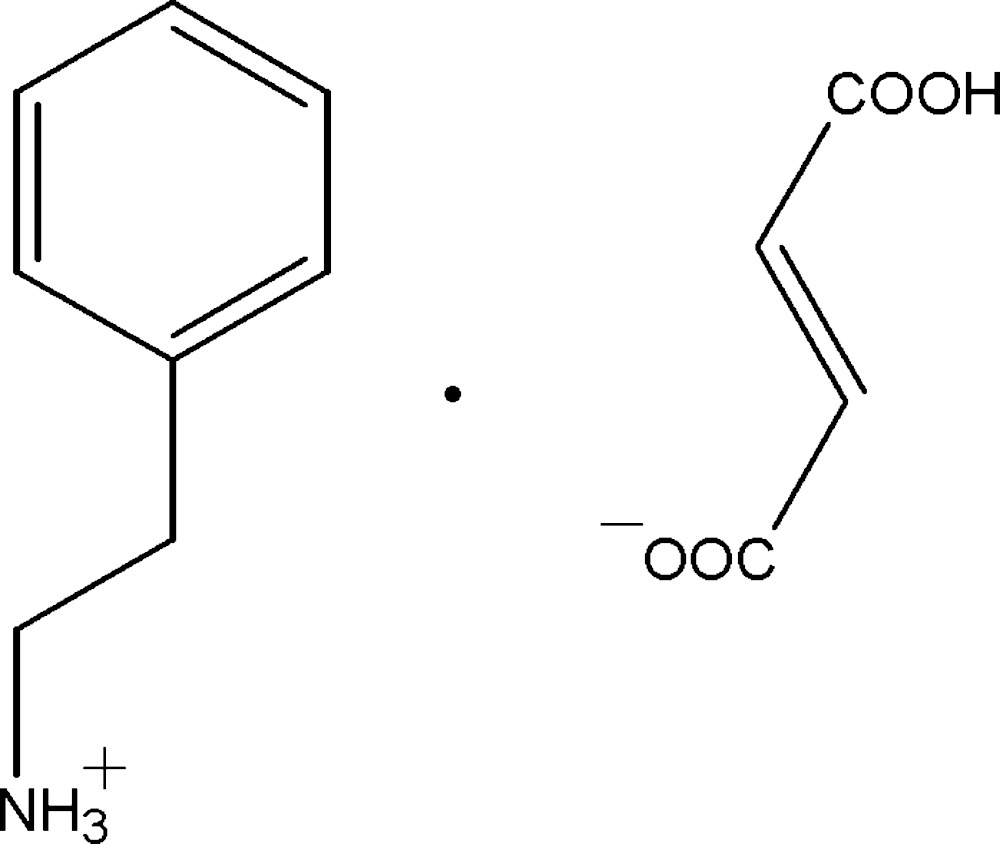


## Experimental   

### Crystal data  

C_8_H_12_N^+^·C_4_H_3_O_4_^−^
*M* *_r_* = 237.25
Triclinic, 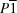

*a* = 9.2940 (5) Å
*b* = 10.8010 (7) Å
*c* = 12.7470 (8) Å
α = 81.773 (4)°
β = 88.907 (5)°
γ = 87.396 (4)°
*V* = 1265.02 (13) Å^3^
*Z* = 4
Mo *K*α radiation
μ = 0.09 mm^−1^
*T* = 295 K
0.26 × 0.24 × 0.20 mm

### Data collection  

Bruker Kappa APEXII CCD diffractometer
Absorption correction: multi-scan (*SADABS*; Sheldrick, 1996 ▸) *T*_min_ = 0.976, *T*_max_ = 0.982
29606 measured reflections
5579 independent reflections
3453 reflections with *I* > 2σ(*I*)
*R*_int_ = 0.039

### Refinement  

*R*[*F* ^2^ > 2σ(*F* ^2^)] = 0.057
*wR*(*F* ^2^) = 0.199
*S* = 1.04
5579 reflections
309 parameters
3 restraints
H-atom parameters constrained
Δρ_max_ = 0.39 e Å^−3^
Δρ_min_ = −0.28 e Å^−3^

### 

Data collection: *APEX2* (Bruker, 2004 ▸); cell refinement: *SAINT* (Bruker, 2004 ▸); data reduction: *SAINT*; program(s) used to solve structure: *SHELXS97* (Sheldrick, 2008 ▸); program(s) used to refine structure: *SHELXL97* (Sheldrick, 2008 ▸); molecular graphics: *PLATON* (Spek, 2009 ▸); software used to prepare material for publication: *SHELXL97* and *PLATON*.

## Supplementary Material

Crystal structure: contains datablock(s) global, I. DOI: 10.1107/S2056989015014292/su5183sup1.cif

Structure factors: contains datablock(s) I. DOI: 10.1107/S2056989015014292/su5183Isup2.hkl

Supporting information file. DOI: 10.1107/S2056989015014292/su5183Isup3.cml

**Fig. 1.**
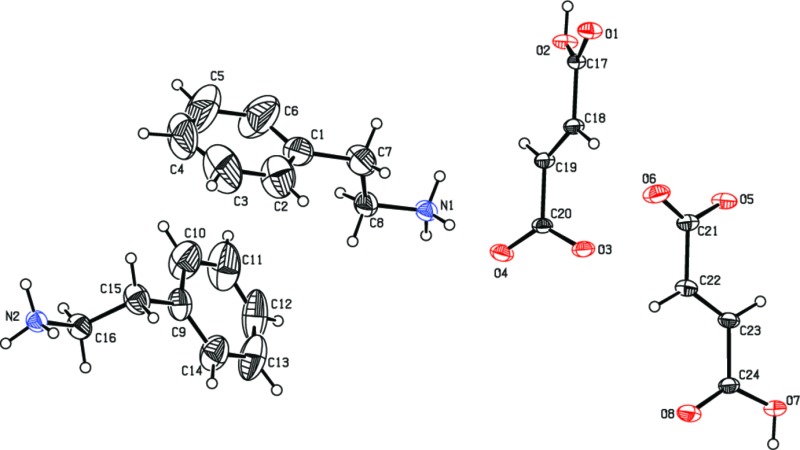
The molecular structure of the title salt, showing the atom labelling. Displacement ellipsoids are drawn at the 30% probability level.

**Fig. 2.**
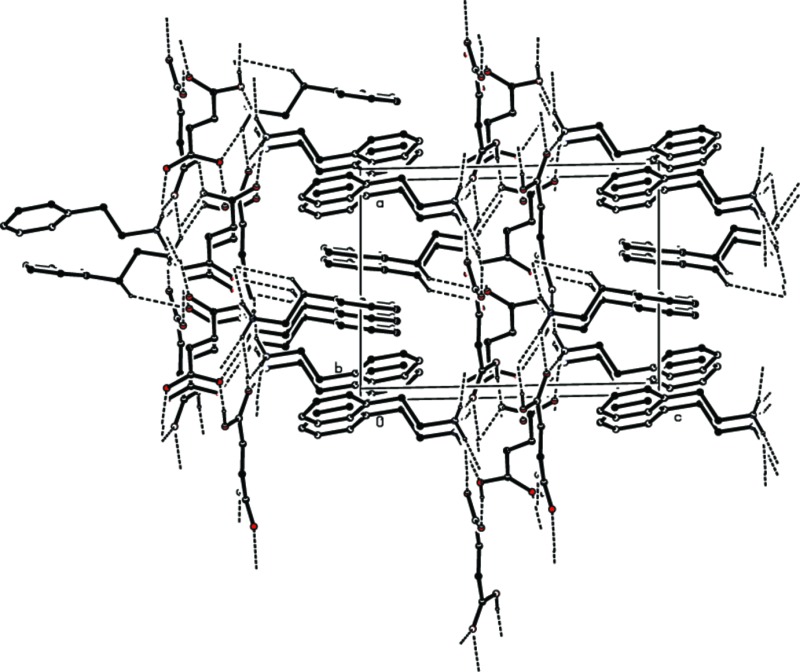
The crystal packing of the title salt viewed along the b axis. The hydrogen bonds are shown as dashed lines (see Table 1) and C-bound H atoms have been omitted for clarity.

. DOI: 10.1107/S2056989015014292/su5183fig1.tif
The mol­ecular structure of the title salt, showing the atom labelling. Displacement ellipsoids are drawn at the 30% probability level.

b . DOI: 10.1107/S2056989015014292/su5183fig2.tif
The crystal packing of the title salt viewed along the *b* axis. The hydrogen bonds are shown as dashed lines (see Table 1) and C-bound H atoms have been omitted for clarity.

CCDC reference: 1415628

Additional supporting information:  crystallographic information; 3D view; checkCIF report

## Figures and Tables

**Table 1:** Hydrogen-bond geometry (, )

*D*H*A*	*D*H	H*A*	*D* *A*	*D*H*A*
N1H1*C*O4	0.89	1.92	2.805(3)	177
N1H1*A*O8^i^	0.89	2.00	2.863(3)	163
N1H1*B*O1^ii^	0.89	2.38	2.992(2)	126
N1H1*B*O5^iii^	0.89	2.20	2.961(3)	143
N2H2*A*O1^iv^	0.89	1.92	2.814(3)	177
N2H2*C*O6^iv^	0.89	1.99	2.863(3)	167
N2H2*B*O7^v^	0.89	2.23	2.974(3)	141
N2H2*B*O4^vi^	0.89	2.38	2.992(2)	126
O2H2*D*O5^vii^	0.82	1.65	2.470(2)	176
O7H7O3^viii^	0.82	1.68	2.494(2)	174
C7H7*A*O8^i^	0.97	2.58	3.349(4)	136

Symmetry codes: (i) 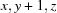; (ii) 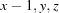; (iii) 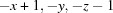; (iv) 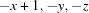; (v) 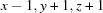; (vi) 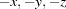; (vii) 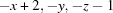; (viii) 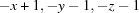.

## supplementary crystallographic information

### S1. Comment

The molecular structure of the title molecular salt is illustrated in Fig. 1. The geometric parameters are comparable with those reported for similar structures (Ambalatharasu *et al.*, 2014; Lejon *et al.*, 2006; Smith *et al.*, 2003). The asymmetric unit consists of two independent 2-phenyl­ethanaminium cations and 3-carb­oxy­prop-2-enoate anions. The cations are protonated at amine-N atoms, and in the cations the side chains exhibit anti-conformations [C1—C7—C8—N1 = 176.5 (3) ° and C9—C15—C16—N2 = 179.4 (3) °]. The dihedral angle between the aromatic rings, (C1—C6) and (C9—C14), is 34.0 (3)Å.

In the crystal, the molecular structure is stabilized by a medium-strength intra­molecular cation-anion N—H···O hydrogen bond (Table 1 and Fig. 2). Adjacent anions and cations are linked by further N—H···O and O—H···O hydrogen bonds into infinite two-dimensional networks parallel to the *ab* plane (Table 1 and Fig. 2). There are also weak C—H···O hydrogen bonds within the sheets (Table 1).

### S2. Synthesis and crystallization

The title slat was synthesized by mixing 2-phenyl­ethyl­amine (1.26 g) and fumaric acid (1.16 g) in methanol-water (1:1) and the single crystals suitable for X-ray diffraction were grown by slow evaporation.

### S3. Refinement

Crystal data, data collection and structure refinement details are summarized in Table 2. All the H atoms were positioned geometrically and refined using a riding model: O—H = 0.82 Å, N—H = 0.89 Å, C—H = 0.93 - 0.97 Å with U_iso_(H) = 1.5U_eq_(O,N) for OH and NH_3_ H atoms and 1.2U_eq_(C) for other H atoms. The bond distances C1—C6, C3—C4 and C5—C6 were restrained to 1.390 (1) Å.

### Crystal data

**Table:**

C_8_H_12_N^+^·C_4_H_3_O_4_^−^	*Z* = 4
*M**_r_* = 237.25	*F*(000) = 504
Triclinic, *P*1	*D*_x_ = 1.246 Mg m^−^^3^
Hall symbol: -P 1	Mo *K*α radiation, λ = 0.71073 Å
*a* = 9.2940 (5) Å	Cell parameters from 7705 reflections
*b* = 10.8010 (7) Å	θ = 2.2–27.1°
*c* = 12.7470 (8) Å	µ = 0.09 mm^−^^1^
α = 81.773 (4)°	*T* = 295 K
β = 88.907 (5)°	Block, colourless
γ = 87.396 (4)°	0.26 × 0.24 × 0.20 mm
*V* = 1265.02 (13) Å^3^	

### Data collection

**Table:**

Bruker Kappa APEXII CCD diffractometer	5579 independent reflections
Radiation source: fine-focus sealed tube	3453 reflections with *I* > 2σ(*I*)
Graphite monochromator	*R*_int_ = 0.039
ω and φ scan	θ_max_ = 27.2°, θ_min_ = 1.9°
Absorption correction: multi-scan (*SADABS*; Sheldrick, 1996)	*h* = −11→11
*T*_min_ = 0.976, *T*_max_ = 0.982	*k* = −13→13
29606 measured reflections	*l* = −16→16

### Refinement

**Table:**

Refinement on *F*^2^	Primary atom site location: structure-invariant direct methods
Least-squares matrix: full	Secondary atom site location: difference Fourier map
*R*[*F*^2^ > 2σ(*F*^2^)] = 0.057	Hydrogen site location: inferred from neighbouring sites
*wR*(*F*^2^) = 0.199	H-atom parameters constrained
*S* = 1.04	*w* = 1/[σ^2^(*F*_o_^2^) + (0.0955*P*)^2^ + 0.6291*P*] where *P* = (*F*_o_^2^ + 2*F*_c_^2^)/3
5579 reflections	(Δ/σ)_max_ < 0.001
309 parameters	Δρ_max_ = 0.39 e Å^−^^3^
3 restraints	Δρ_min_ = −0.28 e Å^−^^3^

### Special details

**Table:**

Geometry. All esds (except the esd in the dihedral angle between two l.s. planes) are estimated using the full covariance matrix. The cell esds are taken into account individually in the estimation of esds in distances, angles and torsion angles; correlations between esds in cell parameters are only used when they are defined by crystal symmetry. An approximate (isotropic) treatment of cell esds is used for estimating esds involving l.s. planes.
Refinement. Refinement of F^2^ against ALL reflections. The weighted R-factor wR and goodness of fit S are based on F^2^, conventional R-factors R are based on F, with F set to zero for negative F^2^. The threshold expression of F^2^ > 2sigma(F^2^) is used only for calculating R-factors(gt) etc. and is not relevant to the choice of reflections for refinement. R-factors based on F^2^ are statistically about twice as large as those based on F, and R- factors based on ALL data will be even larger.

### Fractional atomic coordinates and isotropic or equivalent isotropic displacement parameters (Å^2^)

**Table:**

	*x*	*y*	*z*	*U*_iso_*/*U*_eq_	
C1	0.3953 (4)	0.2508 (4)	−0.0808 (3)	0.0901 (13)	
C2	0.3882 (6)	0.1650 (6)	0.0060 (4)	0.1224 (18)	
H2	0.3988	0.0810	−0.0024	0.147*	
C3	0.3664 (7)	0.1939 (8)	0.1061 (4)	0.159 (3)	
H3	0.3691	0.1307	0.1639	0.191*	
C4	0.3403 (8)	0.3176 (9)	0.1216 (5)	0.175 (4)	
H4	0.3165	0.3389	0.1881	0.210*	
C5	0.3508 (11)	0.4049 (10)	0.0362 (5)	0.214 (4)	
H5	0.3414	0.4888	0.0453	0.257*	
C6	0.3750 (9)	0.3752 (5)	−0.0654 (4)	0.175 (3)	
H6	0.3777	0.4386	−0.1230	0.210*	
C7	0.4238 (4)	0.2131 (5)	−0.1903 (3)	0.0956 (14)	
H7A	0.4901	0.2704	−0.2290	0.115*	
H7B	0.4695	0.1299	−0.1824	0.115*	
C8	0.2922 (3)	0.2139 (3)	−0.2520 (2)	0.0587 (8)	
H8A	0.2434	0.2957	−0.2558	0.070*	
H8B	0.2285	0.1529	−0.2152	0.070*	
C9	−0.0810 (4)	0.2157 (3)	0.0186 (3)	0.0697 (9)	
C10	−0.0453 (7)	0.3273 (4)	−0.0371 (4)	0.1176 (18)	
H10	−0.0028	0.3866	−0.0030	0.141*	
C11	−0.0731 (8)	0.3531 (5)	−0.1475 (4)	0.142 (2)	
H11	−0.0477	0.4290	−0.1859	0.171*	
C12	−0.1362 (7)	0.2677 (6)	−0.1965 (4)	0.124 (2)	
H12	−0.1611	0.2872	−0.2674	0.149*	
C13	−0.1632 (6)	0.1557 (6)	−0.1439 (4)	0.1197 (19)	
H13	−0.1986	0.0946	−0.1798	0.144*	
C14	−0.1388 (5)	0.1290 (4)	−0.0354 (3)	0.0941 (13)	
H14	−0.1619	0.0514	0.0009	0.113*	
C15	−0.0560 (4)	0.1892 (4)	0.1377 (3)	0.0710 (9)	
H15A	−0.0280	0.1014	0.1571	0.085*	
H15B	0.0224	0.2383	0.1556	0.085*	
C16	−0.1874 (3)	0.2197 (3)	0.1996 (2)	0.0553 (7)	
H16A	−0.2159	0.3073	0.1793	0.066*	
H16B	−0.2654	0.1700	0.1819	0.066*	
C17	0.9242 (2)	0.0767 (2)	−0.40812 (17)	0.0297 (5)	
C18	0.8125 (2)	−0.0199 (2)	−0.39499 (18)	0.0323 (5)	
H18	0.8431	−0.1039	−0.3845	0.039*	
C19	0.6736 (2)	0.0079 (2)	−0.39746 (18)	0.0314 (5)	
H19	0.6431	0.0919	−0.4074	0.038*	
C20	0.5617 (2)	−0.0890 (2)	−0.38511 (17)	0.0312 (5)	
C21	0.8888 (2)	−0.3850 (2)	−0.43107 (19)	0.0347 (5)	
C22	0.7711 (2)	−0.4760 (2)	−0.42610 (19)	0.0365 (5)	
H22	0.7233	−0.4982	−0.3619	0.044*	
C23	0.7326 (2)	−0.5258 (2)	−0.5096 (2)	0.0352 (5)	
H23	0.7801	−0.5022	−0.5737	0.042*	
C24	0.6170 (2)	−0.61804 (19)	−0.50667 (19)	0.0336 (5)	
N1	0.3190 (2)	0.1850 (2)	−0.36129 (16)	0.0433 (5)	
H1A	0.3817	0.2375	−0.3943	0.065*	
H1B	0.2366	0.1933	−0.3966	0.065*	
H1C	0.3547	0.1067	−0.3585	0.065*	
N2	−0.1646 (2)	0.1957 (2)	0.31577 (16)	0.0439 (5)	
H2A	−0.1254	0.1189	0.3334	0.066*	
H2B	−0.2488	0.2021	0.3495	0.066*	
H2C	−0.1058	0.2515	0.3341	0.066*	
O1	1.04844 (16)	0.04879 (15)	−0.37585 (14)	0.0410 (4)	
O2	0.88262 (16)	0.18467 (14)	−0.45441 (13)	0.0388 (4)	
H2D	0.9498	0.2318	−0.4584	0.058*	
O3	0.60245 (17)	−0.19778 (15)	−0.40468 (15)	0.0428 (4)	
O4	0.43731 (16)	−0.05906 (15)	−0.35813 (15)	0.0436 (4)	
O5	0.92154 (17)	−0.33377 (15)	−0.52644 (14)	0.0421 (4)	
O6	0.9469 (2)	−0.36439 (18)	−0.34987 (15)	0.0531 (5)	
O7	0.59639 (17)	−0.65424 (16)	−0.59847 (14)	0.0446 (4)	
H7	0.5322	−0.7045	−0.5928	0.067*	
O8	0.54969 (18)	−0.65294 (16)	−0.42533 (14)	0.0459 (5)	

### Atomic displacement parameters (Å^2^)

**Table:**

	*U*^11^	*U*^22^	*U*^33^	*U*^12^	*U*^13^	*U*^23^
C1	0.093 (3)	0.133 (4)	0.049 (2)	−0.040 (3)	0.0008 (18)	−0.020 (2)
C2	0.131 (4)	0.163 (5)	0.068 (3)	0.013 (4)	−0.012 (3)	−0.005 (3)
C3	0.155 (6)	0.270 (10)	0.044 (3)	−0.002 (6)	−0.010 (3)	0.007 (4)
C4	0.144 (5)	0.322 (13)	0.080 (4)	−0.031 (7)	0.014 (4)	−0.098 (6)
C5	0.302 (11)	0.240 (10)	0.131 (6)	−0.054 (8)	0.012 (7)	−0.116 (7)
C6	0.287 (10)	0.155 (6)	0.094 (4)	−0.065 (6)	−0.012 (5)	−0.041 (4)
C7	0.070 (2)	0.161 (4)	0.061 (2)	−0.027 (3)	−0.0009 (18)	−0.029 (2)
C8	0.0557 (17)	0.076 (2)	0.0464 (17)	−0.0103 (15)	0.0040 (13)	−0.0135 (14)
C9	0.082 (2)	0.080 (2)	0.0449 (18)	0.0080 (19)	0.0043 (16)	−0.0078 (17)
C10	0.197 (6)	0.087 (3)	0.068 (3)	−0.021 (3)	0.015 (3)	−0.005 (2)
C11	0.242 (7)	0.099 (4)	0.073 (3)	0.021 (4)	0.035 (4)	0.017 (3)
C12	0.183 (6)	0.136 (5)	0.047 (2)	0.078 (4)	−0.006 (3)	−0.014 (3)
C13	0.154 (5)	0.143 (5)	0.070 (3)	0.037 (4)	−0.025 (3)	−0.049 (3)
C14	0.125 (4)	0.098 (3)	0.062 (2)	−0.003 (3)	−0.005 (2)	−0.021 (2)
C15	0.064 (2)	0.096 (3)	0.0515 (19)	0.0026 (18)	0.0002 (15)	−0.0069 (18)
C16	0.0506 (16)	0.074 (2)	0.0401 (15)	−0.0053 (14)	−0.0039 (12)	−0.0041 (14)
C17	0.0272 (11)	0.0323 (11)	0.0316 (12)	−0.0103 (9)	0.0020 (9)	−0.0085 (9)
C18	0.0310 (11)	0.0294 (11)	0.0374 (13)	−0.0104 (9)	−0.0003 (9)	−0.0054 (9)
C19	0.0292 (11)	0.0284 (11)	0.0375 (12)	−0.0084 (9)	−0.0008 (9)	−0.0050 (9)
C20	0.0271 (11)	0.0356 (12)	0.0320 (12)	−0.0116 (9)	−0.0032 (9)	−0.0046 (9)
C21	0.0315 (11)	0.0289 (11)	0.0441 (14)	−0.0078 (9)	−0.0032 (10)	−0.0042 (10)
C22	0.0335 (12)	0.0340 (12)	0.0419 (13)	−0.0116 (9)	0.0010 (10)	−0.0016 (10)
C23	0.0286 (11)	0.0304 (11)	0.0472 (14)	−0.0102 (9)	0.0034 (10)	−0.0049 (10)
C24	0.0279 (11)	0.0275 (11)	0.0467 (14)	−0.0069 (9)	−0.0003 (10)	−0.0075 (10)
N1	0.0332 (10)	0.0536 (13)	0.0438 (12)	−0.0058 (9)	−0.0007 (9)	−0.0078 (10)
N2	0.0374 (11)	0.0566 (13)	0.0385 (12)	−0.0057 (10)	−0.0005 (9)	−0.0087 (10)
O1	0.0283 (8)	0.0406 (9)	0.0546 (11)	−0.0102 (7)	−0.0051 (7)	−0.0052 (8)
O2	0.0294 (8)	0.0324 (8)	0.0538 (11)	−0.0136 (6)	−0.0033 (7)	0.0006 (7)
O3	0.0306 (8)	0.0359 (9)	0.0656 (12)	−0.0113 (7)	0.0020 (8)	−0.0169 (8)
O4	0.0277 (8)	0.0411 (9)	0.0630 (12)	−0.0097 (7)	0.0057 (7)	−0.0088 (8)
O5	0.0384 (9)	0.0423 (9)	0.0452 (10)	−0.0192 (7)	−0.0041 (7)	0.0014 (8)
O6	0.0586 (11)	0.0563 (11)	0.0474 (11)	−0.0278 (9)	−0.0094 (9)	−0.0081 (9)
O7	0.0358 (9)	0.0456 (10)	0.0569 (11)	−0.0192 (7)	0.0046 (8)	−0.0177 (8)
O8	0.0420 (10)	0.0457 (10)	0.0510 (11)	−0.0205 (8)	0.0058 (8)	−0.0055 (8)

### Geometric parameters (Å, º)

**Table:**

C1—C2	1.341 (6)	C15—H15A	0.9700
C1—C6	1.3894 (10)	C15—H15B	0.9700
C1—C7	1.524 (5)	C16—N2	1.484 (3)
C2—C3	1.366 (8)	C16—H16A	0.9700
C2—H2	0.9300	C16—H16B	0.9700
C3—C4	1.3888 (10)	C17—O1	1.243 (3)
C3—H3	0.9300	C17—O2	1.276 (3)
C4—C5	1.339 (11)	C17—C18	1.496 (3)
C4—H4	0.9300	C18—C19	1.312 (3)
C5—C6	1.3902 (10)	C18—H18	0.9300
C5—H5	0.9300	C19—C20	1.499 (3)
C6—H6	0.9300	C19—H19	0.9300
C7—C8	1.465 (5)	C20—O4	1.242 (3)
C7—H7A	0.9700	C20—O3	1.276 (3)
C7—H7B	0.9700	C21—O6	1.229 (3)
C8—N1	1.485 (4)	C21—O5	1.298 (3)
C8—H8A	0.9700	C21—C22	1.498 (3)
C8—H8B	0.9700	C22—C23	1.322 (3)
C9—C10	1.360 (6)	C22—H22	0.9300
C9—C14	1.373 (5)	C23—C24	1.495 (3)
C9—C15	1.524 (4)	C23—H23	0.9300
C10—C11	1.421 (7)	C24—O8	1.222 (3)
C10—H10	0.9300	C24—O7	1.306 (3)
C11—C12	1.347 (8)	N1—H1A	0.8900
C11—H11	0.9300	N1—H1B	0.8900
C12—C13	1.329 (8)	N1—H1C	0.8900
C12—H12	0.9300	N2—H2A	0.8900
C13—C14	1.393 (6)	N2—H2B	0.8900
C13—H13	0.9300	N2—H2C	0.8900
C14—H14	0.9300	O2—H2D	0.8200
C15—C16	1.491 (4)	O7—H7	0.8200
			
C2—C1—C6	116.4 (5)	C16—C15—H15A	109.2
C2—C1—C7	121.5 (5)	C9—C15—H15A	109.2
C6—C1—C7	122.1 (4)	C16—C15—H15B	109.2
C1—C2—C3	123.8 (6)	C9—C15—H15B	109.2
C1—C2—H2	118.1	H15A—C15—H15B	107.9
C3—C2—H2	118.1	N2—C16—C15	112.6 (2)
C2—C3—C4	120.0 (6)	N2—C16—H16A	109.1
C2—C3—H3	120.0	C15—C16—H16A	109.1
C4—C3—H3	120.0	N2—C16—H16B	109.1
C5—C4—C3	116.9 (6)	C15—C16—H16B	109.1
C5—C4—H4	121.6	H16A—C16—H16B	107.8
C3—C4—H4	121.6	O1—C17—O2	124.44 (18)
C4—C5—C6	122.7 (8)	O1—C17—C18	119.9 (2)
C4—C5—H5	118.6	O2—C17—C18	115.61 (18)
C6—C5—H5	118.6	C19—C18—C17	123.3 (2)
C1—C6—C5	119.9 (6)	C19—C18—H18	118.4
C1—C6—H6	120.0	C17—C18—H18	118.4
C5—C6—H6	120.0	C18—C19—C20	123.3 (2)
C8—C7—C1	112.8 (3)	C18—C19—H19	118.4
C8—C7—H7A	109.0	C20—C19—H19	118.4
C1—C7—H7A	109.0	O4—C20—O3	124.52 (19)
C8—C7—H7B	109.0	O4—C20—C19	119.1 (2)
C1—C7—H7B	109.0	O3—C20—C19	116.41 (19)
H7A—C7—H7B	107.8	O6—C21—O5	125.11 (19)
C7—C8—N1	113.5 (3)	O6—C21—C22	120.8 (2)
C7—C8—H8A	108.9	O5—C21—C22	114.1 (2)
N1—C8—H8A	108.9	C23—C22—C21	122.5 (2)
C7—C8—H8B	108.9	C23—C22—H22	118.8
N1—C8—H8B	108.9	C21—C22—H22	118.8
H8A—C8—H8B	107.7	C22—C23—C24	123.6 (2)
C10—C9—C14	118.2 (4)	C22—C23—H23	118.2
C10—C9—C15	120.1 (4)	C24—C23—H23	118.2
C14—C9—C15	121.7 (3)	O8—C24—O7	125.07 (19)
C9—C10—C11	119.9 (5)	O8—C24—C23	121.8 (2)
C9—C10—H10	120.0	O7—C24—C23	113.2 (2)
C11—C10—H10	120.0	C8—N1—H1A	109.5
C12—C11—C10	120.0 (5)	C8—N1—H1B	109.5
C12—C11—H11	120.0	H1A—N1—H1B	109.5
C10—C11—H11	120.0	C8—N1—H1C	109.5
C13—C12—C11	120.3 (5)	H1A—N1—H1C	109.5
C13—C12—H12	119.9	H1B—N1—H1C	109.5
C11—C12—H12	119.9	C16—N2—H2A	109.5
C12—C13—C14	120.5 (5)	C16—N2—H2B	109.5
C12—C13—H13	119.8	H2A—N2—H2B	109.5
C14—C13—H13	119.8	C16—N2—H2C	109.5
C9—C14—C13	120.9 (5)	H2A—N2—H2C	109.5
C9—C14—H14	119.6	H2B—N2—H2C	109.5
C13—C14—H14	119.6	C17—O2—H2D	109.5
C16—C15—C9	111.8 (3)	C24—O7—H7	109.5
			
C6—C1—C2—C3	−1.7 (8)	C10—C9—C14—C13	−1.4 (7)
C7—C1—C2—C3	178.4 (5)	C15—C9—C14—C13	178.9 (4)
C1—C2—C3—C4	4.4 (10)	C12—C13—C14—C9	−2.7 (8)
C2—C3—C4—C5	−5.9 (12)	C10—C9—C15—C16	94.4 (5)
C3—C4—C5—C6	5.1 (14)	C14—C9—C15—C16	−85.9 (4)
C2—C1—C6—C5	0.7 (10)	C9—C15—C16—N2	−179.4 (3)
C7—C1—C6—C5	−179.3 (6)	O1—C17—C18—C19	159.4 (2)
C4—C5—C6—C1	−2.6 (14)	O2—C17—C18—C19	−21.4 (3)
C2—C1—C7—C8	100.0 (5)	C17—C18—C19—C20	179.5 (2)
C6—C1—C7—C8	−79.9 (6)	C18—C19—C20—O4	159.5 (2)
C1—C7—C8—N1	176.5 (3)	C18—C19—C20—O3	−21.2 (3)
C14—C9—C10—C11	2.3 (8)	O6—C21—C22—C23	−161.6 (2)
C15—C9—C10—C11	−178.0 (5)	O5—C21—C22—C23	18.0 (3)
C9—C10—C11—C12	0.8 (9)	C21—C22—C23—C24	179.2 (2)
C10—C11—C12—C13	−4.9 (10)	C22—C23—C24—O8	0.3 (4)
C11—C12—C13—C14	5.9 (9)	C22—C23—C24—O7	179.6 (2)

### Hydrogen-bond geometry (Å, º)

**Table:**

*D*—H···*A*	*D*—H	H···*A*	*D*···*A*	*D*—H···*A*
N1—H1*C*···O4	0.89	1.92	2.805 (3)	177
N1—H1*A*···O8^i^	0.89	2.00	2.863 (3)	163
N1—H1*B*···O1^ii^	0.89	2.38	2.992 (2)	126
N1—H1*B*···O5^iii^	0.89	2.20	2.961 (3)	143
N2—H2*A*···O1^iv^	0.89	1.92	2.814 (3)	177
N2—H2*C*···O6^iv^	0.89	1.99	2.863 (3)	167
N2—H2*B*···O7^v^	0.89	2.23	2.974 (3)	141
N2—H2*B*···O4^vi^	0.89	2.38	2.992 (2)	126
O2—H2*D*···O5^vii^	0.82	1.65	2.470 (2)	176
O7—H7···O3^viii^	0.82	1.68	2.494 (2)	174
C7—H7*A*···O8^i^	0.97	2.58	3.349 (4)	136

Symmetry codes: (i) *x*, *y*+1, *z*; (ii) *x*−1, *y*, *z*; (iii) −*x*+1, −*y*, −*z*−1; (iv) −*x*+1, −*y*, −*z*; (v) *x*−1, *y*+1, *z*+1; (vi) −*x*, −*y*, −*z*; (vii) −*x*+2, −*y*, −*z*−1; (viii) −*x*+1, −*y*−1, −*z*−1.
