# Supplementary figures and images for: Crystal structure of 2-phenyl­ethanaminium 3-carb­oxy­prop-2-enoate
Source: Acta Crystallogr E Crystallogr Commun. 2015 Aug 6;71(Pt 9):o641–2. doi: 10.1107/S2056989015014292 (PMC4555434; doi:10.1107/S2056989015014292)

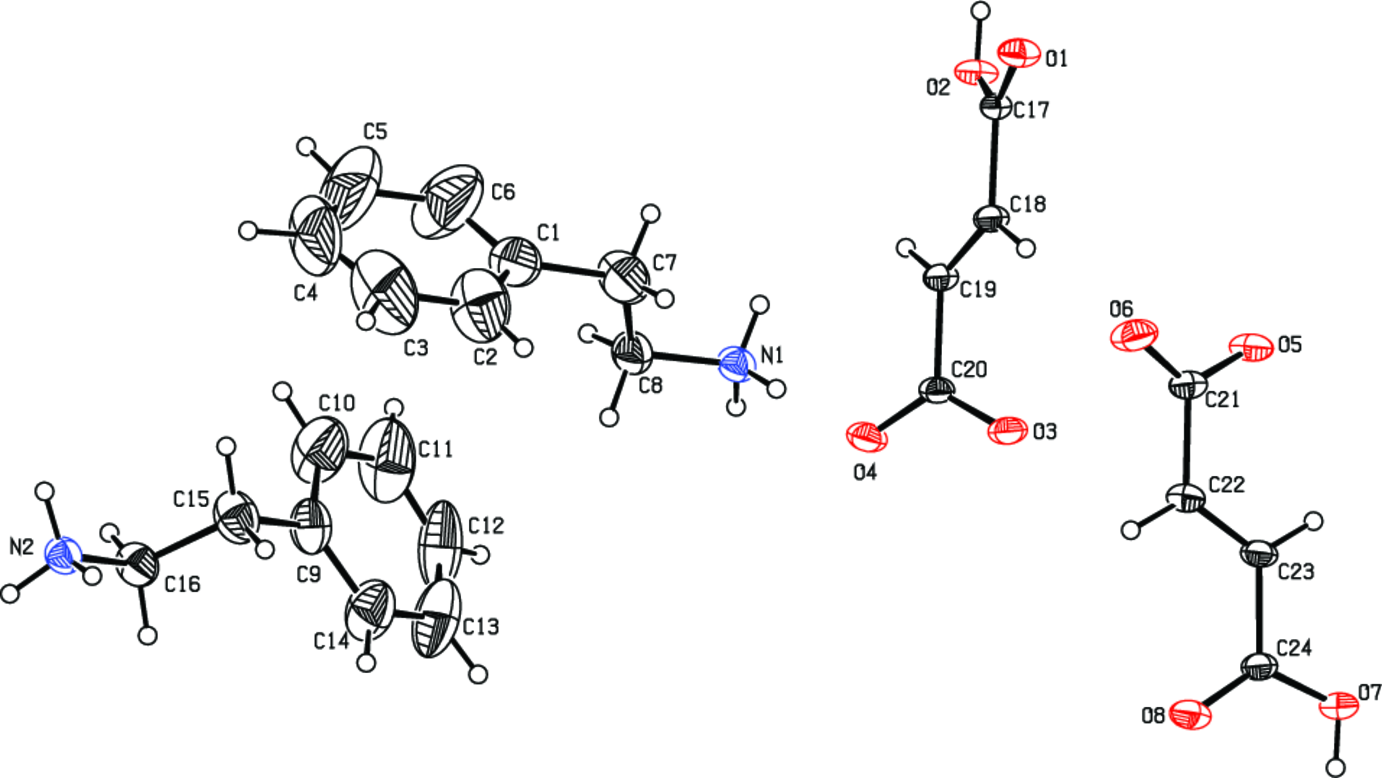

Supplement: Supplementary file 4 [file e-71-0o641-fig1.tif]

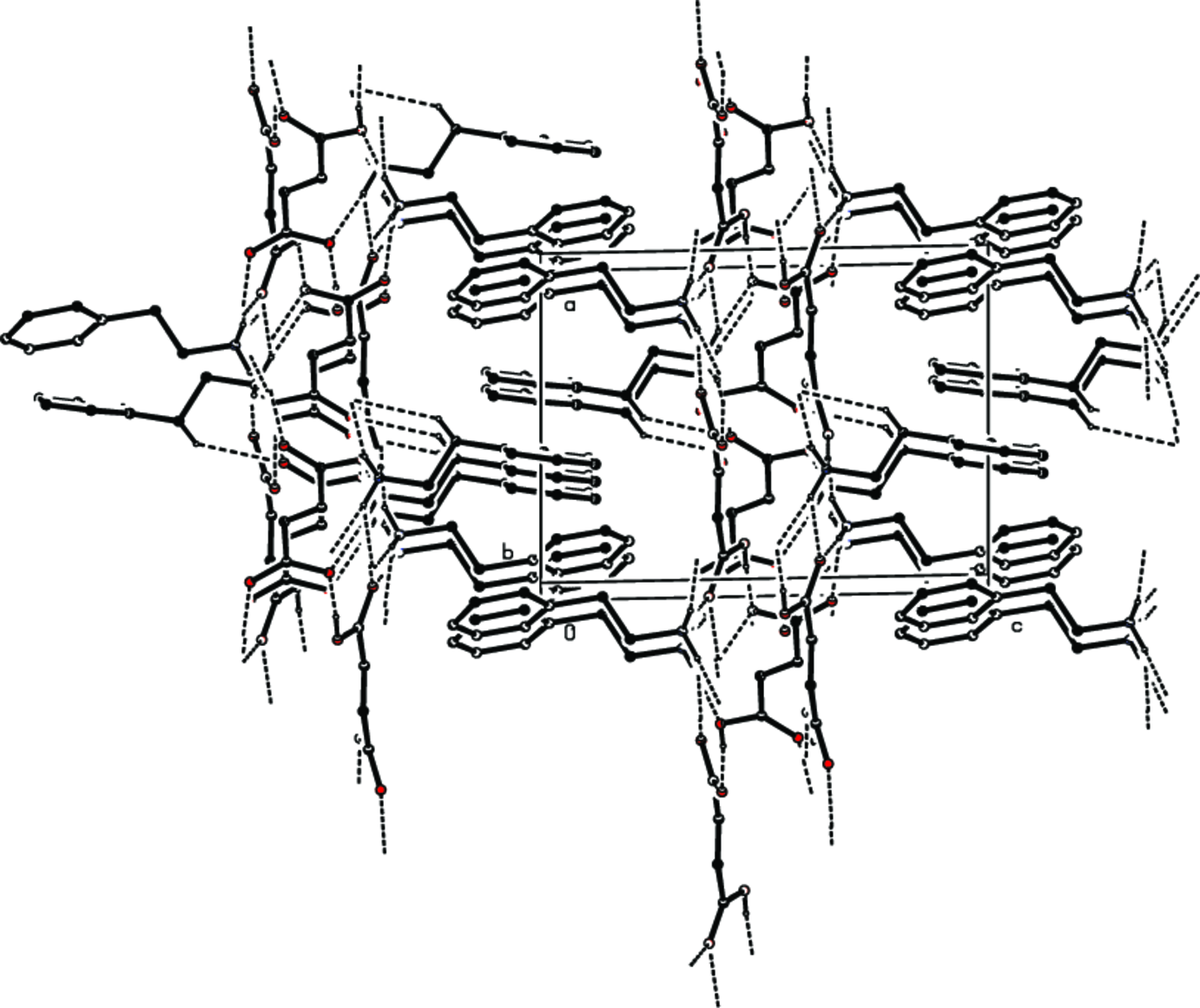

Supplement: Supplementary file 5 [file e-71-0o641-fig2.tif]
